# Clinical and Angiographic Profiles of Myocardial Infarction in a Young South Indian Population

**DOI:** 10.7759/cureus.63949

**Published:** 2024-07-06

**Authors:** Surya Prakash, Joy M Thomas, Rajaram Anantharaman

**Affiliations:** 1 Interventional Cardiology, Panimalar Medical College Hospital & Research Institute, Chennai, IND; 2 Interventional Cardiology, Apollo Hospitals, Chennai, IND; 3 Interventional Cardiology, Kauvery Hospital, Chennai, IND

**Keywords:** types 2 diabetes, coronary artery disease, angiographic profiles, clinical profiles, south indians, young adults, myocardial infarction

## Abstract

Introduction

Myocardial infarction (MI) in young South Indians presents a shifting epidemiological landscape, challenging traditional perceptions of cardiovascular diseases. This study investigates the clinical and angiographic profiles of MI in this subgroup of the population in detail, emphasizing the interaction between lifestyle, environmental, and genetic factors that contribute to the incidence of MI in younger people.

Methodology

Utilizing a descriptive observational design, the study analyzed data from 70 young adults (aged 18-45 years) admitted to Frontier Lifeline Hospital, Chennai, with acute MI over six months. Patient demographics, clinical characteristics, and angiographic findings were collected and analyzed using standardized protocols. Statistical analysis employed chi-square tests and subgroup analyses to assess associations and differences between diabetic and non-diabetic patients.

Results

The study revealed a predominance of males (84.29%) among MI cases, with ST-elevation myocardial infarction (STEMI) being the most common presentation (52.86%). Anterior wall involvement was prevalent (50%), and left ventricular systolic dysfunction (LVSD) was observed in the majority (67%) of patients. Chest pain (87%) was the predominant symptom, and diabetes (47%) and hypertension (47%) were the risk variables that were most common. Angiographically, the left anterior descending artery (LAD) was often affected (51%), with single-vessel disease predominating (41.43%).

Conclusion

The findings underscore the significance of early detection and intervention strategies for MI in young South Indians. Gender-specific risk assessment, prompt diagnosis, and tailored treatment approaches are imperative. The high prevalence of LVSD highlights the burden of cardiac morbidity, particularly in diabetic individuals. Lifestyle modifications and weight management interventions are crucial for MI prevention. This study provides insights into the frequency and features of MI in young South Indians, emphasizing the importance of collaborative efforts for early identification and control of modifiable risk factors to mitigate the burden of coronary artery disease (CAD) in this population subset.

## Introduction

Myocardial infarction (MI) is a catastrophic cardiovascular occurrence that happens due to an abrupt reduction in blood supply to the heart muscle, resulting in tissue impairment and potentially fatal consequences [1,2]. Historically, MI has been perceived as a malady primarily afflicting older adults, often associated with atherosclerosis and other age-related cardiovascular conditions. However, in recent decades, a concerning shift in demographics has been observed, with a noticeable increase in the incidence of MI among younger individuals [3].

The epidemiological landscape of MI in young Indians presents a compelling narrative of shifting health paradigms. Traditionally regarded as a disease of aging, MI's emergence in younger age groups challenges preconceived notions and underscores the dynamic interplay of genetic, environmental, and lifestyle factors shaping cardiovascular health [4,5]. Epidemiological data indicate a notable uptick in MI incidence among individuals below the age of 45 in South India, surpassing trends observed in other geographical regions. This epidemiological shift warrants meticulous investigation to discern the underlying drivers fuelling this alarming trend and devise targeted interventions to mitigate its impact [6,7].

Understanding the clinical spectrum of MI in young South Indians is paramount for timely diagnosis and effective management. While chest pain remains the hallmark symptom of MI, young adults may present with atypical manifestations, including dyspnea, and palpitations, or even exhibit asymptomatic forms of the condition [8]. The nuanced clinical presentation poses diagnostic challenges, often leading to delays in seeking medical attention and initiating appropriate interventions. Moreover, sociocultural factors and psychosocial stressors prevalent in South Indian society may influence symptom perception and healthcare-seeking behavior, further complicating the clinical landscape.

A crucial part of understanding the anatomical basis of MI and directing treatment choices is angiographic evaluation [9]. Research on young South Indians has revealed unique angiographic patterns with a tendency for the left anterior descending artery (LAD) involvement and a higher frequency of single-vessel illness [10]. However, the presence of multi-vessel disease is not uncommon, particularly in individuals harboring significant risk factor burdens [11]. Exploring these angiographic profiles offers valuable insights into the pathophysiological mechanisms underpinning MI in young South Indians and informs tailored treatment strategies aimed at optimizing patient outcomes.

The risk factor landscape associated with MI in young South Indians is multifaceted, encompassing a complex interplay of genetic predispositions, lifestyle choices, and socioeconomic determinants. Genetic susceptibility, compounded by the adoption of sedentary lifestyles, unhealthy dietary habits, tobacco use, and psychosocial stressors, amplifies the risk of premature MI in this population subset. Addressing these modifiable risk factors through targeted interventions holds immense promise in curbing the burgeoning burden of MI and fostering cardiovascular health promotion initiatives tailored to the unique needs of young South Indians.

Moreover, delineating the specific risk factors associated with MI in young individuals enhances our understanding of the diverse etiologies contributing to this condition. Gulati et al. classified acute MI in the young population based on risk factors, including traditional cardiovascular risk factors akin to those seen in older individuals, substance abuse, MI due to spontaneous coronary artery dissection (SCAD), myocarditis, or coronary embolism (CE), MI due to atheromatous coronary artery disease (CAD) sans critical coronary stenosis, and coronary vasospasm [12]. Traditional risk factors for CAD in the young, such as plaque rupture, account for a significant proportion of acute MI cases [13]. Understanding these risk factors provides crucial insights into preventive strategies and therapeutic interventions tailored to the unique needs of young South Indians.

## Materials and methods

This study utilizes a descriptive observational design to explore the clinical and angiographic profiles of MI in young adults admitted to Frontier Lifeline Hospital, a tertiary cardiac hospital in Chennai, over a period of six months between January 2020 and June 2020. All patients admitted to the coronary care unit (CCU) with a diagnosis of acute MI, aged 18-45 years, were included. The study excludes patients above 45 years and those unwilling to participate. Following approval from the Institutional Ethics Committee (IEC) and Institutional Research Committee (IRC) from the Institutional Ethics Committee (IEC)of Frontier Lifeline Hospital (ref. no. FLL/IEC/04/2019), patient demographic details, clinical characteristics, and angiographic findings are collected using a standardized proforma. Investigations such as blood profiles, electrocardiograms, echocardiograms, and coronary angiograms were performed, and data were tabulated for statistical analysis.

The clinical profile assessment focuses on demographic characteristics, presenting symptoms, medical history, cardiovascular risk factors, and in-hospital outcomes. Parameters evaluated include age, gender distribution, prevalence of risk factors (hypertension, diabetes, dyslipidemia, and smoking), atypical symptomatology, and short-term clinical outcomes. Angiographic profiles are evaluated using coronary angiography reports to determine the distribution, severity, and complexity of CAD. Data on diseased vessels, culprit lesion location, significant stenosis, and involvement of specific coronary arteries were documented. Plaque morphology, coronary vasospasm, and other angiographic abnormalities contributing to MI pathogenesis were analyzed.

Statistical analysis, performed using Stata Statistical Software release 11.2 (StataCorp LLC, StataCorp., College Station, TX), employs chi-square tests for goodness of fit to assess associations between ACS subtypes, left ventricular dysfunction, and coronary artery involvement. Demographic characteristics, ACS events, symptoms, and risk factors are described as frequencies and percentages. Subgroup analyses were conducted among diabetic and non-diabetic individuals, with results expressed likewise. A significance level of p < 0.05 guides interpretation.

## Results

The age distribution of the 70 patients in the study group varied by age group, with the age range of 41-45 years old accounting for the majority (61.43%). The presentation age ranged from 26 to 45 years old, with a mean of 40.93 years. In terms of gender distribution, males experienced AMI at a higher rate (84.29%) than females (15.71%).

The most prevalent presentation of acute coronary syndrome (ACS) events was found to be ST-segment elevation myocardial infarction (STEMI), which accounted for 52.86% of cases. Non-STEMI (NSTEMI) came in second with 38.57%, while unstable angina (UA) came in third with 8.57%. The most prevalent manifestation of MI, according to the territorial distribution of cases, was anterior wall involvement (50%), which was followed by inferior wall involvement (31.43%) (Table 1).

**Table 1:** Age-wise distribution of myocardial infarction in age <45 years ACS: acute coronary syndrome, STEMI: ST-elevation myocardial infarction, NSTEMI: non-ST-elevation myocardial infarction, UA: unstable angina

Age-wise distribution:		
Variable	Number of cases	Percentage
26-30	2	2.86%
31-35	6	8.57%
36-40	19	27.14%
41-45	43	61.43%
Total	70	
Sex distribution:		
Variable	Number of cases	Percentage
Male	59	84.29%
Female	11	15.71%
Total	70	
ACS events:		
Variable	Number of cases	Percentage
NSTEMI	27	38.57%
STEMI	37	52.86%
UA	6	8.57%
Total	70	

In 67% of the patients, LVSD was found, albeit to varied degrees of severity. Killip class I accounted for the majority of presentations (68.57%), with class II (22.86%), class III (5.71%), and class IV (2.86%) following closely behind. The mean duration from symptom onset to hospital admission was 1.87 days, with patients presenting within a wide range of 1 hour to 10 days.

Symptomatically, chest pain was the predominant symptom observed in 87% of the study population, followed by breathlessness (53%) and autonomic symptoms (64%). The most prevalent risk factors included diabetes (47%), hypertension (47%), dyslipidemia (45%), and smoking (36%). In addition, a subset of patients had a prior history of MI (13%) and a family history of premature CAD (24%) (Table 2).

**Table 2:** Clinical presentation and risk factors in the young South Indian study population with myocardial infarction BMI: body mass index, CAD: coronary artery  disease, MI: myocardial infarction

Category	Subcategory	Number of cases	Percentage
Symptom at presentation	Chest pain		
	Yes	61	87%
	No	9	13%
	Total	70	100%
	Breathlessness		
	Yes	37	53%
	No	33	47%
	Total	70	100%
	Autonomic symptoms		
	Yes	45	64%
	No	25	36%
	Total	70	100%
Risk factors	Diabetes		
	Yes	33	47%
	No	37	53%
	Total	70	100%
	Hypertension		
	Yes	33	47%
	No	37	53%
	Total	70	100%
	Dyslipidemia		
	Yes	31	45%
	No	38	55%
	Total	70	100%
	Smoking		
	Yes	25	36%
	No	45	64%
	Total	70	100%
	Prior MI		
	Yes	9	13%
	No	61	87%
	Total	70	100%
	Family H/O premature CAD		
	Yes	17	24%
	No	53	76%
	Total	70	100%
	BMI		
	Normal	21	30.00%
	Overweight	29	41.43%
	Obese	20	28.57%
	Moderate	18	25.71%
	Severe	1	1.43%
	Very Severe	1	1.43%
	Total	70	100%
Killip class	Total number of risk factors		
	0	6	8.57%
	1	5	7.14%
	2	11	21.43%
	3	22	31.43%
	4	13	18.57%
	5	5	7.14%
	6	2	2.86%
	7	2	2.86%
	Total	70	100%

The most prevalent types of vascular involvement were single-vessel disease (41.43%), double-vessel disease (31.43%), and triple-vessel disease (21.43%). The most often implicated arteries were the left anterior descending artery (LAD) (51%), right coronary artery (RCA) (54%), and left circumflex artery (LCX) (37%). Only 11% of the patients had ectatic coronaries, and there were no instances of spontaneous coronary artery dissections (SCAD) (Table 3, Table 4).

**Table 3:** Territorial distribution of MI MI: myocardial infarction, RV: right ventricle

	Number of cases	Percentage
Anterolateral wall	3	4.29%
Anterior	35	50%
Inferolateral	1	1.43%
Inferoposterior	4	5.72%
Inferoposterior + RV	2	2.86%
Inferior	22	31.43%
Lateral	3	4.29%
Total	70	

**Table 4:** Angiographic profiles of the young south Indian study population with myocardial infarction SVD: single-vessel disease, DVD: double-vessel disease, TVD: triple-vessel disease, LAD: left anterior descending artery, LCX: left circumflex artery, RCA: right coronary artery, ACS: acute coronary syndrome, STEMI: ST-elevation myocardial infarction, NSTEMI: non-ST-elevation myocardial infarction, UA: unstable angina, MI: myocardial Infarction

Category	Subcategory	Number of cases	Percentage
Vessel involvement	SVD	29	41.43%
	DVD	22	31.43%
	TVD	15	21.43%
	Non-obstructive	3	4.29%
	Total	70	100%
Coronary artery involved	LMCA	7	10%
	Proximal LAD	36	51%
	Mid LAD	30	43%
	LCX	26	37%
	RCA	37	54%
	Total	70	100%
Others	Ectatic coronaries	8	11%
	No ectatic coronaries	62	89%
	Total	70	100%

Our subgroup analysis comparing clinical and angiographic parameters between diabetic and non-diabetic patients provided insightful findings regarding the differential presentation and severity of MI in these groups. While no significant differences were observed in ACS events and coronary artery involvement, diabetic patients exhibited a higher prevalence of LVSD and a propensity for more severe vessel involvement, highlighting the complex interplay between diabetes and MI pathophysiology.

Our data specifically showed that 88% of the diabetes individuals had chest discomfort, with just 12% of patients without this symptom. Furthermore, compared to 59% of non-diabetic individuals, 70% of diabetes patients had autonomic symptoms such as palpitations and sweating. With 58% and 49% of patients with diabetes and non-diabetes, respectively, STEMI was the most frequent presentation. Furthermore, LV dysfunction was observed in 58% of patients with diabetes and 49% of participants without the disease.

In addition, our results indicated that 26% of the diabetes individuals had triple vascular disease, 38% double-vessel disease, and 35% single-vessel disease. By contrast, triple vascular disease affected 20% of non-diabetic individuals, double-vessel disease affected 29%, and single-vessel disease affected 51%. These findings highlight how crucial it is to maximize glycemic management in diabetics in order to reduce the risk of unfavorable cardiac outcomes (Table 5).

**Table 5:** Comparison of clinical parameters between the diabetic and non-diabetic young South Indian study population with myocardial infarction SVD: single-vessel disease, DVD: double-vessel disease, TVD: triple-vessel disease, LAD: left anterior descending artery, LCX: left circumflex artery, RCA: right coronary artery, ACS: acute coronary syndrome, STEMI: ST-elevation myocardial infarction, NSTEMI: non-ST-elevation myocardial infarction, UA: unstable angina, MI: myocardial Infarction

Category	Subcategory	Diabetes	No diabetes	Total	P-value
Chest pain	Yes	29 (88%)	32 (86%)	61	0.862
	No	4 (12%)	5 (14%)	9	
	Total	33	37	70	
Autonomic symptoms	Yes	23 (70%)	22 (59%)	45	0.372
	No	10 (30%)	15 (41%)	25	
	Total	33	37	70	
ACS events	NSTEMI	11 (33%)	16 (43%)	27	0.695
	STEMI	19 (58%)	18 (49%)	37	
	UA	3 (9%)	3 (8%)	6	
	Total	33	37	70	
LV dysfunction	Normal	8 (24%)	8 (22%)	16	0.529
	Adequate	6 (18%)	11 (30%)	17	
	LV Dysfunction	19 (58%)	18 (49%)	37	
	Total	33	37	70	
Coronary artery involvement	LMCA	3 (9%)	4 (11%)	7	0.811
	Proximal LAD	18 (54%)	18 (49%)	36	0.622
	Mid LAD	15 (45%)	15 (42%)	30	0.751
	LCX	15 (45%)	11 (31%)	26	0.202
	RCA	18 (54%)	19 (53%)	37	0.883
	Total	33	37	70	
Number of vessel involved	SVD	11 (35%)	18 (51%)	29	0.427
	DVD	12 (38%)	10 (29%)	22	
	TVD	8 (26%)	7 (20%)	15	
	Total	33	37	70	

## Discussion

Our research on MI among young South Indians has yielded important insights into the angiographic and clinical characteristics of this demographic subgroup. The present discourse delves into the consequences of our findings and their importance concerning the management and preventive tactics of MI.

**Figure 1:**
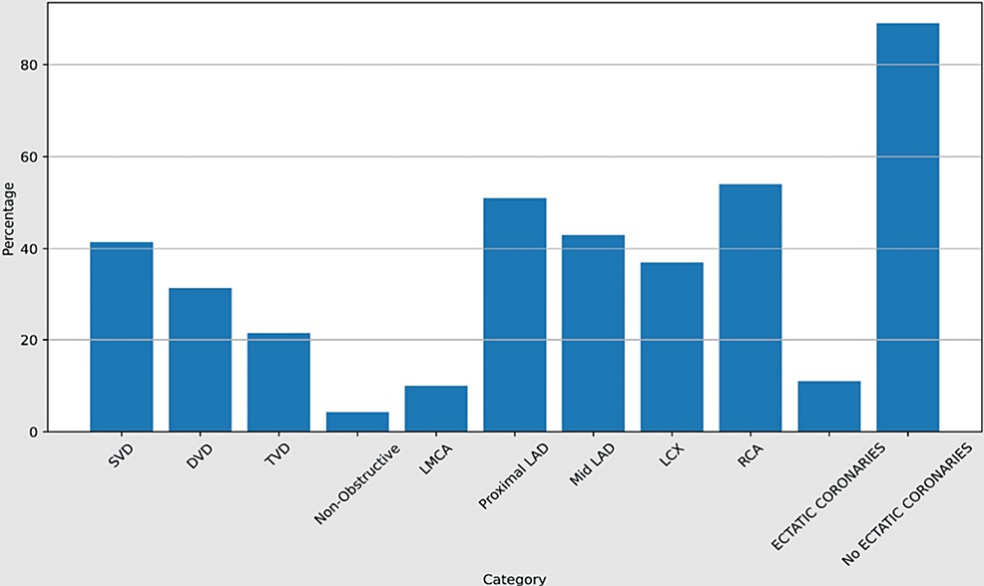
Angiographic profiles of young south indian study population with myocardial infarction SVD: single-vessel disease, DVD: double-vessel disease, TVD: triple-vessel disease, LMCA: left main coronary artery, LAD: left anterior descending artery, LCX: left circumflex artery, RCA: right coronary artery, ACS: acute coronary syndrome, STEMI: ST-elevation myocardial infarction, NSTEMI: non-ST-elevation myocardial infarction, UA: unstable angina

First, our study revealed a notable prevalence of MI among young South Indians, with a majority of cases falling within the age range of 41-45 years. This observation underscores the importance of early detection and intervention strategies targeting this demographic group. Despite being traditionally considered a disease of the elderly, the rising incidence of MI among younger individuals necessitates heightened awareness and preventive measures tailored to this age group (Figure 1).

The majority of MI patients in our analysis were men, which is consistent with the body of research showing that men are more likely than women to have CAD. However, the large percentage of female patients emphasizes how crucial gender-specific risk assessment and management techniques are for preventing MI.

In terms of ACS events, our study identified STEMI as the most common presentation, followed by NSTEMI and UA. This distribution emphasizes the critical role of timely diagnosis and intervention, particularly in cases of STEMI where prompt reperfusion therapy can significantly improve outcomes. Furthermore, the varying territorial distribution of MI underscores the heterogeneity of CAD manifestations and necessitates tailored treatment approaches based on the site of myocardial involvement.

The high prevalence of LVSD among MI patients in our study is concerning, indicating a substantial burden of cardiac morbidity in this population subset. The association between diabetes and LVSD highlights the need for aggressive risk factor modification and targeted management strategies in diabetic individuals to mitigate the risk of adverse cardiac outcomes.

The research now in publication is supported by the existence of conventional risk factors such as smoking, diabetes, hypertension, dyslipidemia, and hypertension; nevertheless, the high incidence of obesity and overweight in young South Indians highlights the growing significance of metabolic variables in MI etiology. This finding underscores the importance of lifestyle modifications and weight management interventions in MI prevention.

**Figure 2:**
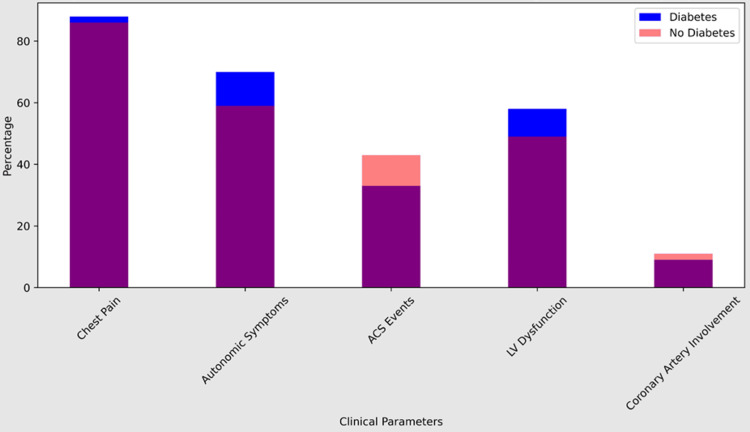
Comparison of clinical parameters between the diabetic and non-diabetic young South Indian study population with myocardial infarction ACS: acute coronary syndrome, LV: left ventricle

**Figure 3:**
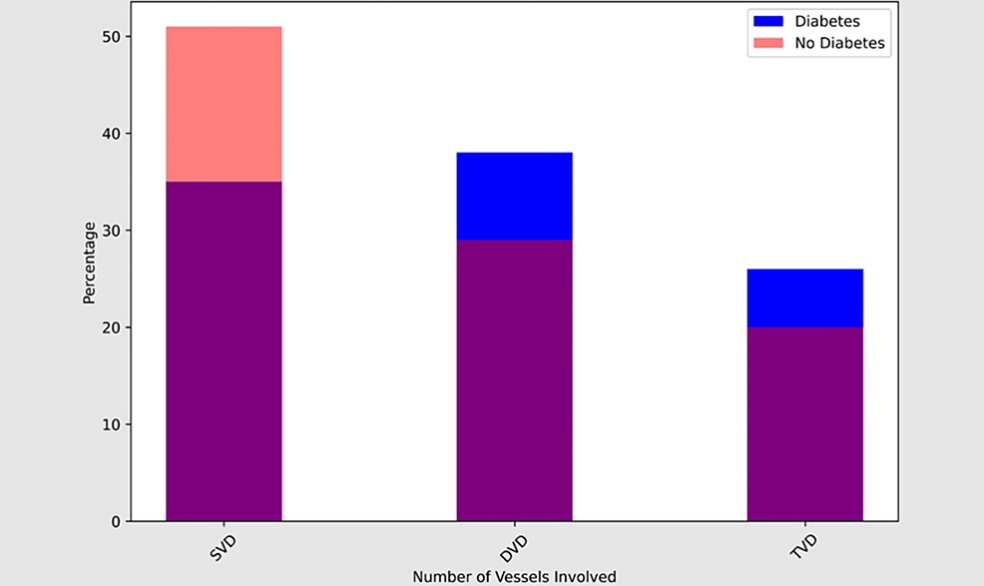
Comparison of the number of vessels involved between the diabetic and non-diabetic young South Indian study population SVD: single-vessel disease, DVD: double-vessel disease, TVD: triple-vessel disease

Our subgroup analysis comparing clinical and angiographic parameters between diabetic and non-diabetic patients yielded valuable insights into the differential presentation and severity of MI in these groups. While no significant differences were observed in ACS events and coronary artery involvement, diabetic patients exhibited a higher prevalence of LVSD and a propensity for more severe vessel involvement, highlighting the complex interplay between diabetes and MI pathophysiology (Figures 2, 3).

Furthermore, the association between glycemic control parameters and MI severity underscores the importance of optimizing glycemic control in diabetic individuals to mitigate the risk of adverse cardiac outcomes. This finding emphasizes the need for comprehensive risk factor management and multidisciplinary care approaches in MI prevention and management among young South Indians. Overall, our study contributes to the growing body of evidence on MI epidemiology and risk factors in young South Indians, providing valuable insights for clinicians and policymakers in developing targeted interventions and preventive strategies tailored to this population subset.

The investigation into AMI among young South Indians draws insights from multiple studies, each shedding light on different aspects of this condition. The study by Deshmukh et al. highlights the predominance of male patients and the high prevalence of anterior wall myocardial infarction (AWMI), frequently attributed to LAD involvement, among 41 patients under 30 years with AMI [14].

Within the 300 young patients with STEMI included in the study by Pandya et al., smoking was found to be a significant risk factor, which highlights the importance of prompt diagnosis and treatment [15]. Another study by Rawat observed a high prevalence of male gender and LAD involvement in obstructive CAD cases among 104 AMI patients under 35 years [16].

Similarly, the study by Joshi et al. focused on 22 AMI patients ≤30 years, revealing less extensive CAD and a high prevalence of smoking as a risk factor [17]. Another study by Kumbhalkar and Bisne highlighted dyslipidemia and smoking as major risk factors and single-vessel disease as the most prevalent angiographic finding among 70 cases of young IHD [18]. The study by Ashida et al. in a tertiary cardiac hospital observed smoking as the predominant risk factor and single-vessel disease, particularly involving LAD, as a common angiographic finding among 104 young patients with STEMI [19].

In the study by Prakash et al., dyslipidemia, smoking, and diabetes mellitus were identified as major risk factors, with left anterior descending artery involvement being common among 224 young ACS patients [20]. Finally, among 117 young CAD patients, the study by Pandey et al. in a tier II city of Eastern India observed a predominance of men and single-vessel disease, particularly involving the left anterior descending artery [21]. Together, these studies underscore the multifaceted nature of AMI in young South Indians, emphasizing the importance of early detection, risk factor management, and tailored interventions to improve outcomes in this population subset.

## Conclusions

Our study highlights the prevalence and characteristics of acute myocardial infarction (AMI) in youth, with a higher incidence in males. Chest pain was the most typical manifestation, regardless of the existence of diabetes. STEMI was the most common acute coronary syndrome event. Risk factors such as obesity, diabetes mellitus, and systemic hypertension were often mentioned and were indicative of changing lifestyles and urbanization. Angiographically, single-vessel disease involvement was more prevalent, with the LAD being the most affected. The ACS presentations of individuals with and without diabetes were similar, although the former had more severe LVSD and a higher frequency of triple- or double-vessel disease. In the younger population, collaborative efforts are needed for the primary prevention of CAD. The limitation of our study is it is a small group analysis, and hence a multicentered study involving a large population is needed.
